# Anesthesia management of thoracic giant tumor resection: two case reports

**DOI:** 10.1093/jscr/rjaf842

**Published:** 2026-01-02

**Authors:** Jinfeng Nie, Xiaoping Wang, Tinghong Chen, Bing Chen, Yunfei Zhang

**Affiliations:** Department of Anesthesia, Liangjiang Hospital of Chongqing Medical University (People’s Hospital of Chongqing Liangjiang New Area), 2 Jin Kai Da Dao, Liangjiang New Area, Chongqing 401121, China; Department of Anesthesia, Liangjiang Hospital of Chongqing Medical University (People’s Hospital of Chongqing Liangjiang New Area), 2 Jin Kai Da Dao, Liangjiang New Area, Chongqing 401121, China; Department of Anesthesia, Liangjiang Hospital of Chongqing Medical University (People’s Hospital of Chongqing Liangjiang New Area), 2 Jin Kai Da Dao, Liangjiang New Area, Chongqing 401121, China; Department of Anesthesia, Liangjiang Hospital of Chongqing Medical University (People’s Hospital of Chongqing Liangjiang New Area), 2 Jin Kai Da Dao, Liangjiang New Area, Chongqing 401121, China; Department of Anesthesia, The Third Affiliated Hospital of Zunyi Medical University (The First People's Hospital of Zunyi), 106 Feng Huang Bei Lu, Zunyi 563000, Guizhou, China

**Keywords:** anesthesia, thoracic tumor, resection

## Abstract

Surgical excision remains the primary therapeutic approach for giant tumors located within the thoracic cavity. However, these tumors frequently induce compression of critical structures such as the airways, lungs, heart, and vena cava, thereby posing significant challenges for perioperative respiratory and hemodynamic management. This report details the anesthetic management strategies employed in two patients undergoing resection of giant thoracic tumors: one patient with dwarfism and another with severe respiratory and circulatory insufficiency. These cases offer important strategies for airway management during general anesthesia, the prevention of recruitment pulmonary edema, and the facilitation of rapid postoperative recovery in this complex patient population.

## Introduction

Surgical intervention is considered the most effective modality to enhance quality of life in patients presenting with large thoracic tumors characterized by prolonged growth duration and indolent progression [1]. Nevertheless, the rarity of such cases and the complexity of perioperative respiratory and circulatory management render anesthetic care particularly challenging [2]. This article presents the anesthetic management of two successful resections of giant thoracic tumors.

## Case 1

**Figure 1:**
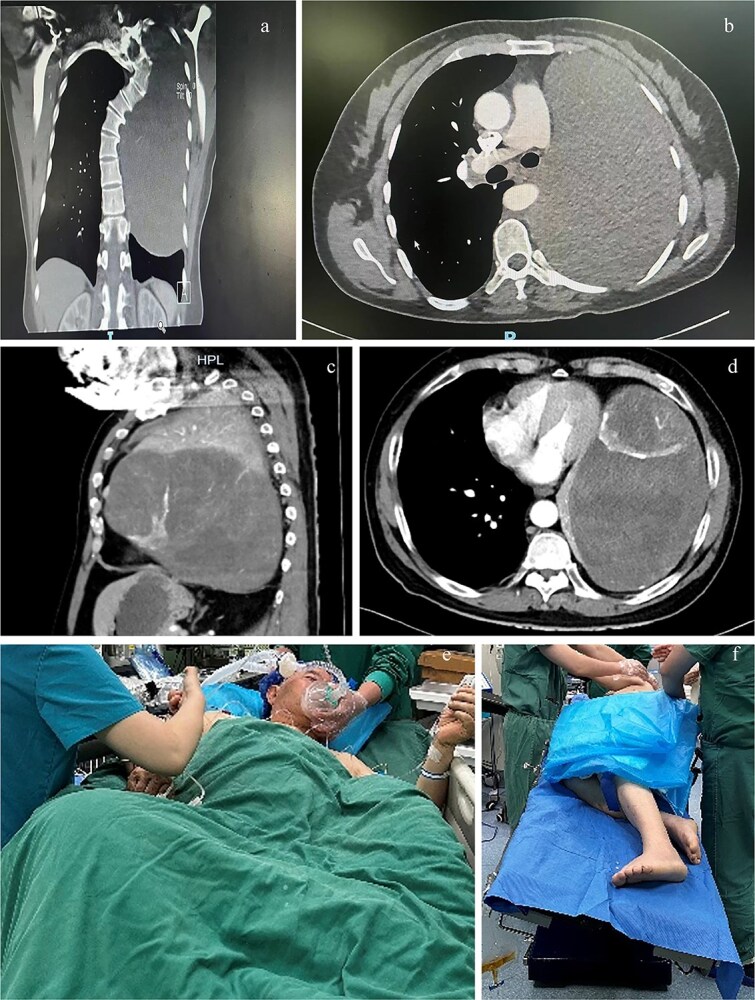
Computed tomography (CT) planes of case 1 in (a) coronal and (b) transverse planes and case 2 in (c) sagittal and (d) transverse planes. The (e) semirecumbent left-tilted posture of case 2 during anesthesia induction and the (f) right lateral decubitus position with the operating bed tilted leftward of case 2 during surgery.

A 48-year-old female patient with 140 cm and 41 kg was admitted with a 1-month history of cough, sputum production, and wheezing. Computed tomography (CT) revealed a solid mass measuring 10.7 × 16.0 × 20.5 cm within the left thoracic cavity (Fig. 1a and b). Bronchial angiography and embolization were performed 2 weeks prior to surgery. Pulmonary rehabilitation exercises were initiated.

Airway evaluation indicated no anticipated difficulty with intubation before anesthesia. Ventilation and hemodynamics remained stable in the right lateral decubitus position. Ultrasound-guided radial artery cannulation was performed for invasive blood pressure monitoring, and right internal jugular venous access was established for fluid administration and central venous pressure monitoring. Anesthesia induction was conducted in the supine position using midazolam, etomidate, fentanyl, and vecuronium. Postinduction, fiberoptic bronchoscopy was utilized to measure the distance from the tracheal carina to the incisors (19 cm), guiding the insertion of a 28 French right-sided double-lumen bronchial tube initially advanced to 21 cm. Auscultation of the right upper and lower lung fields guided incremental withdrawal to optimize right main bronchus alignment. Concurrently, the ability to smoothly advance a suction catheter beyond the length of the main tube was used to confirm alignment with the left bronchial orifice. Finally, 19.5 cm was the optimizing tube positioning to ensure appropriate alignment with the right main bronchus and left bronchial orifice.

The patient was gradually repositioned to the right lateral decubitus position, with immediate repositioning in response to hypotension. A restrictive fluid management protocol guided by invasive blood pressure and urine output was employed, supplemented by continuous norepinephrine infusion to maintain circulatory stability. Colloid solutions were primarily administered before tumor resection, transitioning to transfusion of red blood cells (RBCs) and fresh frozen plasma (FFP) after blood loss exceeded 1000 ml. Furosemide 10 mg was given prior to lung recruitment maneuvers. Nasopharyngeal temperature was maintained above 36°C throughout the procedure. The surgery lasted 6 hours. Intraoperative fluid management included a 500 ml crystalloid, a 1500 ml colloid, a 1600 ml RBC suspension, and 1400 ml FFP. Estimated blood loss was 3000 ml, with urine output of 600 ml. Two minutes following lung recruitment, 20 ml of pink fluid was aspirated from the airway; no further similar secretions were observed after 1 hour. Extubation was successfully performed on postoperative day 1, with discharge occurring on day 12.

## Case 2

A 74-year-old male patient with height 172 cm and weight 74 kg presented with a left thoracic mass identified 7 years prior. Over the preceding 6 months, he experienced abdominal distension, anorexia, wheezing, recurrent lower limb edema, and orthopnea. CT demonstrated a solid mass measuring 11.1 × 19.2 × 23.0 cm occupying the left thoracic cavity (Fig. 1c and d), with rightward cardiac displacement. Vascular embolization was not achieved for the inability to identify the tumor’s feeding vessels and the patient’s intolerance of prolonged supine positioning.

The patient was positioned comfortably in a semirecumbent left-tilted posture (Fig. 1e) during anesthesia. Anesthesia induction was achieved via slow-inhalational administration of sevoflurane (incrementally increased to 5%) combined with intermittent boluses of propofol (1 mg), maintaining spontaneous ventilation. Airway topical anesthesia was administered using the spray-as-you-go technique [3]. A 37 French right-sided double-lumen bronchial tube was inserted and positioned under fiberoptic bronchoscopy guidance ~15 minutes later. The patient was then transferred to the operating bed and gradually repositioned to the right lateral decubitus position with the operating bed tilted leftward (Fig. 1f). Subsequent anesthetic management mirrored that of case 1. The surgery lasted 7 hours. Intraoperative blood loss was 8000 ml, necessitating transfusion of 27 units of RBCs, 5000 ml FFP, one therapeutic dose of platelets, 180 g albumin, 1000 ml of the crystalloid, and 4500 ml of the colloid. Urine output was 550 ml. Following lung recruitment, 30 ml of pink fluid was observed in the airway over a 10-minute period. The patient was extubated on postoperative day 3 and discharged on day 16.

## Discussion

The impact of surgical positioning on respiratory and circulatory function must be assessed preoperatively to tailor individualized induction strategies. In case 1, the tumor exerted minimal influence on respiratory and circulatory parameters in the right lateral decubitus position, permitting conventional induction in the supine position. Conversely, in case 2, tumor compression precluded supine positioning, necessitating induction techniques that preserved spontaneous ventilation to avoid exacerbation of airway and circulatory compromise [4]. In addition, gradual repositioning postinduction is essential to prevent abrupt respiratory or hemodynamic instability.

Lung isolation via double-lumen bronchial intubation is the preferred airway management technique to prevent contamination of the healthy lung. Accurate placement of double-lumen tubes requires fiberoptic bronchoscopy or bilateral lung auscultation. However, in patients with large thoracic tumors who require the smallest available double-lumen tubes—such as pediatric patients or adults with short stature (case 1)—fiberoptic bronchoscopes cannot pass through the tube lumen, and auscultation may be unreliable. Case 1 employed a novel dual-chamber bronchial tube positioning method to address this challenge. This approach may offer a viable alternative for patients in whom conventional fiberoptic or auscultatory methods are impractical for double-lumen tube positioning.

Intraoperative management necessitates vigilant hemodynamic monitoring and judicious volume administration to prevent hypotension, volume overload, and recurrent pulmonary edema secondary to circulatory insufficiency [5]. Fluid resuscitation prioritized colloids, albumin, and blood products to maintain oncotic pressure, supplemented by vasoactive agents to sustain hemodynamic stability. Diuretic therapy was administered prior to lung recruitment maneuvers. Strict intraoperative temperature control was maintained to preserve internal homeostasis. Although mild pulmonary edema occurred, it was transient and resolved promptly.

In summary, the experiences described herein provide valuable clinical guidance for anesthetic care in patients with dwarfism or severe respiratory and circulatory insufficiency undergoing resection of giant pleural tumors.
